# Lack of MMP-9 expression is a marker for poor prognosis in Dukes’ B colorectal cancer

**DOI:** 10.1186/1472-6890-12-24

**Published:** 2012-12-07

**Authors:** Selja Koskensalo, Jaana Hagström, Nina Linder, Mikael Lundin, Timo Sorsa, Johanna Louhimo, Caj Haglund

**Affiliations:** 1 Department of Surgery, Helsinki University Central Hospital, P.O. Box 440, 00029 HUS, Helsinki, Finland; 2 Department of Pathology, Haartman Institute, University of Helsinki, Helsinki, FIN-00014 HY, Finland; 3 Institute for Molecular Medicine Finland (FIMM), University of Helsinki, Helsinki, FIN-00014 HY, Finland; 4 Institute of Dentistry, University of Helsinki and Department of Oral and Maxillofacial Diseases, Helsinki University Central Hospital, Helsinki, Finland

**Keywords:** MMP-2, MMP-8, MMP-9, Colorectal cancer, Prognosis, Immunohistochemistry

## Abstract

**Background:**

Matrix metalloproteinases (MMPs) play a role in cancer progression by degrading extracellular matrix and basement membranes, assisting in tumour neovascularization and in supporting immune response in cancer.

**Methods:**

We studied the prognostic value of immunohistochemical expression of MMP-2, MMP-8, and MMP-9 in a series of 619 colorectal cancer patients using tissue microarray specimens.

**Results:**

Of the samples, 56% were positive for MMP-2, 78% for MMP-8, and 60% for MMP-9. MMP-9 associated with low WHO grade (p < 0.001). In univariate analysis of Dukes’ B tumours, MMP-9 negativity associated with poor survival (p = 0.018), and MMP-9 positivity was an independent prognostic marker in multivariate analysis of these tumours (p = 0.034).

**Conclusion:**

Negative MMP-9 expression can predict poor prognosis in Dukes’ B colorectal tumours and may prove useful for identifying patients, who should be offered adjuvant treatment.

## Background

Colorectal cancer (CRC) is the third most common malignancy in the world [1]. The most important prognostic factor in colorectal cancer is stage of disease at diagnosis. Other factors for poor prognosis are venous and lymphatic invasion, deficient healthy tissue margins at surgery, obstruction or perforation of the bowel, and poor differentiation at histology [2-5]. In addition to tumour-specific factors, also host responses, such as intra- or peritumoral inflammation and desmoplasia or reactive lymph nodes can suppress tumour spread and predict better outcome [6-8]. In cancer progression and spread, tumours must invade their surrounding tissues, basement membranes (BM), and extracellular matrix (ECM), must avoid the host’s immunoresponse, and must ascertain their circulation by neovascularization. In all these phenomena, matrix metalloproteinases (MMPs) play an important role [9].

MMPs are zinc-dependent endopeptidases capable of degrading both BM and ECM proteins and extracellular adhesion molecules. MMP-9, also known as gelatinase B, is able to degrade collagens IV (the main component of BM) and V, gelatins, and elastin. MMP-2, also known as gelatinase A, is able to degrade the same substrates as MMP-9, but also collagens I, VII, X, fibronectin and procollagenase-3 [9]. MMP-8, also called collagenase-2, is able to degrade collagens I, II and III [10].

In CRC, MMP-2 immunoexpression associates with advanced disease [11,12], and high MMP-2 expression in cancer cells and the stroma associates with poor prognosis [11,13]. MMP-9 correlates with metastatic disease [11,14,15] and poor prognosis [14], although conflicting findings exist [16]. MMP-8 is expressed in many cancer types [17,18] and may protect against cancer spread by regulating tumour metastasis [18].

In this study, we studied the prognostic value of MMP-2, MMP-8 and MMP-9 immunoexpression in colorectal cancer.

## Methods

### Patients

Sample material came from 643 consecutive patients who underwent surgery for CRC at the Department of Surgery, Meilahti Hospital, Helsinki University Central Hospital, between 1982 and 1998. Of these, 18 patients were excluded on account of incorrect final diagnosis (9 patients) or synchronous multiple tumours (9 patients). Six cases were excluded because of insufficient archival surgical tissue specimens. Finally, 619 cases remained, 330 of them male. Tumour staging was performed according to the modified Dukes’ classification [19], with 91 tumours classified as Dukes’ stage A, 226 as stage B, 161 as stage C, and 145 as stage D. For patients’ clinicopathological characteristics, see Table 1.

**Table 1:** Patient clinicopathological characteristics and their correlation with MMP-2, MMP-8, and MMP-9 immunoreactivity in colorectal cancer patients assessed with chi-square test

	**MMP-2**				**MMP-8**				**MMP-9**			
**Clinicopathological**	**Patients**	**positive**		***p-value***	**Patients**	**positive**		***p-value***	**Patients**	**positive**		***p-value***
variable	(n=)	(n)	%		(n=)	(n)	%		(n=)	(n)	%	
Gender				0.887				0.496				0.574
Female	272	153	56.3		257	194	75.5		257	158	61.5	
Male	309	172	55.7		301	235	78.1		301	192	63.8	
Age				0.107				0.343				0.197
< 65 years	244	146	59.8		228	183	80.3		238	142	59.7	
≥ 65 years	337	179	53.1		320	246	76.9		320	208	65.0	
Dukes’ stage				0.317				0.148				0.100
A	87	44	50.6		82	63	76.8		83	57	68.7	
B	208	110	52.9		197	163	82.7		206	138	67.0	
C	155	93	60.0		137	99	72.3		138	77	55.8	
D	131	78	59.5		132	104	78.8		131	78	59.5	
Dukes’ stage				0.066				0.116				0.016*
A and B	295	154	52.2		279	226	81.0		289	195	67.5	
C and D	286	171	59.8		269	203	75.5		269	155	57.6	
Differentiation (WHO grade)				0.116				0.326				<0.001*
1	19	9	47.4		19	14	73.7		18	12	66.7	
2	382	211	55.2		368	290	78.8		374	254	67.9	
3	155	86	55.5		138	111	80.4		142	77	54.2	
4	24	19	79.2		22	14	63.6		23	7	30.4	
missing	1				1				1			
Histologic type				0.680				0.085				0.053
Adenocarcinoma	512	288	56.3		481	382	79.4		489	314	64.2	
Mucinous carcinoma	69	37	53.6		67	47	70.1		69	36	52.2	
Tumour location				0.202				0.073				0.419
Colon	315	169	53.7		305	247	81.0		311	191	61.4	
Rectum	263	155	58.9		240	179	74.6		244	158	64.8	
missing	3				3				3			

* = p-value significant.

Clinical data were retrieved from patient records, and survival and cause of death data until March 2011 from the Population Register Centre of Finland, and Statistics Finland.

For MMP-9, we also stained tissue microarray (TMA) specimens from a validation series of 213 CRC patients treated between 1998–2001, 82 of them male; 31 tumours were classified as Dukes’ stage A, 70 stage B, 69 stage C, and 41 stage D, with 201 of tumours being adenocarcinomas; with 7 being grade I, 161 grade II, 37 grade III, and 4 grade IV. Clinical data was retrieved, as for the main cohort, until October 2011.

### Tissue samples and preparation of TMA blocks

Formalin-fixed and paraffin-embedded surgical tissue samples were collected from the archives of the Department of Pathology, University of Helsinki. Histopathologically representative regions of tumour specimens were defined and marked on H&E slides. Three cores from each tumour block were sampled with 1.0 mm punchers with a manual tissue microarrayer (Tissue Arrayer 1, Beecher Instruments Inc., Silver Spring, MD, USA). Three series of blocks were constructed, all including one sample from each patient. From each block, 4-μm slides were cut and processed for immunohistochemistry.

### Immunohistochemistry of MMP-2, MMP-8, and MMP-9

The Lab Vision Autostainer TM 480 (LabVision, Fremont, CA, USA) served for immunohistochemistry. Specimens were deparaffinized in xylene and rehydrated through graded alcohol series. To retrieve antigen, samples were heated in the pretreatment module of the autostainer in Tris–HCl pH 8.5 buffer for 20 minutes at 98°C. The samples were incubated for 5 min in DAKO REAL Peroxidase-Blocking Solution (DAKO, Glostrup, Denmark) for inactivation of endogenous peroxidases. The sections were incubated for one hour with a monoclonal primary MMP-2 antibody MS-806-PO (NeoMarkers) diluted 1:700, with a polyclonal MMP-8 antibody 1:100 [20] or with a polyclonal MMP-9 antibody RB-1539-R7 (NeoMarkers) 1:1500 and reacted for 30 min with the Dako REAL EnVision™/HRP detection system, Rabbit/Mouse (ENV) reagent. Between each pair of steps the sections were rinsed with Tween-20/PBS. The peroxidase staining was visualized with 3-amino-9-ethylcarbatzole (Sigma-Aldrich, Inc., St. Louis, MO, USA). Slides were counterstained with Meyer’s haematoxylin, washed in tap water for 10 min, and mounted in aqueous mounting medium (Aquamount, BDH, Poole, UK). Stainings of specimens without primary MMP-antibody were used as negative controls. Gingival tissue was used as positive control in MMP-8 stainings, and gastric tissue in MMP-2 and MMP-9 stainings.

### Scoring of immunostainings

**Figure 1:**
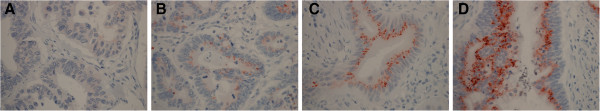
**Immunohistochemical scoring pattern of MMP-9 in colorectal cancer.A** no expression, **B** mild, **C** moderate, **D** strong.

MMP-2, MMP-8, and MMP-9 immunostainings were scored by two independent investigators (S.K. and J.H.) without knowledge of clinical outcome. In case of different scores, the consensus score was determined. The interobserver variation was low, below 5%. The intensity of cytoplasmic staining in cancer cells was evaluated: strong intensity, scored as 3, moderate as 2, and weak as 1. Absence of positivity in all spots was scored as 0 (Figure 1). Spots without cancer cells were excluded. For further analysis, the patients were divided into two groups, negative (0) versus positive (score 1–3).

### Statistical analysis

Correlations between stainings and clinicopathological variables were assessed with the χ2 test or Fisher’s exact test when applicable. The Mann–Whitney U-test was applied to determine the correlation between age and the immunoreactivity. Life-tables were calculated with the Kaplan-Meier method. The significance of the difference between groups was assessed with the log-rank test or log-rank test for trend. Patients alive at the end of the follow-up (March 2011) and patients who died from unrelated causes or postoperatively (within 30 days from surgery) were censored. The Cox proportional hazards model served for multivariate survival analysis. Clinical variables included in the model as covariates were age, Dukes’ stage, differentiation (WHO grade), tumour location (colon or rectum), and tumour histology (adenocarcinoma or mucinous carcinoma).

The likelihood ratio test was applied for exclusion or inclusion of significant variables. A p-value of 0.05 was considered significant. Statistical analyses were performed with SPSS 17.0 software.

## Results

### Tissue expression of MMP-2, MMP-8, and MMP-9

#### Immunostaining for MMP-2

Of 581 cases, MMP-2 immunoreactivity was observed in 325 (55.9%). In 56 patients (9.6%), MMP-2 immunopositivity was scored as strong, in 98 (16.9%) as moderate, and in 171 (29.4%) as weak, whereas 256 (44.1%) lacked any MMP-2 immunopositivity. MMP-2 positivity did not correlate with clinicopathological variables (Table 1).

#### Immunostaining for MMP-8

Of 548 cases, MMP-8 immunoreactivity was noticed in 429 (78.3%). In 39 patients (7.1%), MMP-8 immunopositivity was scored as strong, in 153 (27.9%) as moderate, and in 237 (43.2%) as weak, whereas 119 (21.7%) lacked any MMP-8 immunopositivity. MMP-8 positivity showed no correlation with clinicopathological variables (Table 1).

#### Immunostaining for MMP-9

Of the 581 cases, MMP-9 immunoreactivity was observed in 350 (60.2%). In 50 patients (8.6%), MMP-9 immunopositivity was scored as strong, in 108 (18.6%) as moderate, and in 192 (33.0%) as weak, whereas 208 (35.8%) lacked any MMP-9 immunopositivity. MMP-9 correlated with WHO grade (p < 0.001); it was more often positive in high to moderately differentiated tumours. MMP-9 immunopositivity did not correlate with Dukes’ stage, but was more often positive in local (Dukes’ A-B) tumours than in advanced disease (p = 0.016 ) (Table 1).

In the validation series of 213 cases MMP-9 was positive in 167 (78.4%). In positive patients, in 12 (7.2%), the immunopositivity was scored as strong, in 41 (24.6%) as moderate, and in 114 (68.3%) as weak. No correlation appeared between clinicopathological variables and MMP-9 immunoexpression.

### Prognostic roles of MMP-2, MMP-8, and MMP-9

In univariate analysis, 5-year survival was 62.5% in MMP-9-positive, and 52.2% in MMP-9-negative patients, a difference that was significant (p = 0.015). Advanced Dukes’ stage (p < 0.001), old age (p = 0.005), and higher WHO grade (p < 0.001) associated with poor prognosis (Table 2). The association between elevated MMP-9 immunoexpression and improved prognosis was evident only in Dukes’ B tumours, but it was such a strong prognostic factor that the association was significant in analysis of the whole cohort (p = 0.018). In Dukes’ A stage 5-year survival was 92.3% in MMP-9 positive and 87.1% in MMP-9 negative patients (p = 0.417), in Dukes’ C patients 56.6% versus 50.3% (p = 0.618), and in Dukes’ D patients 6.8% versus 9.6% (p = 0.992).

**Table 2:** Univariate analysis of correlations between preoperative characteristics and survival with Kaplan-Meier life-table and logrank test analyses

**Clinicopathological**	**Patients**		**Cumulative 5-year**	**χ2**	***p-value***
**variable**	**(n)**	**%**	**survival %**	**statistic**	
MMP2 immunoreactivity	581			0.507	0.477
Negative	256	44.1	58.8		
Positive	325	55.9	56.0		
MMP8 immunoreactivity	548			0.129	0.719
Negative	119	21.7	56.6		
Positive	429	78.3	58.8		
MMP9 immunoreactivity	558			5.923	0.015*
Negative	208	44.9	52.2		
Positive	350	55.1	62.5		
Gender	619			0.000	0.992
Female	289	46.7	56.1		
Male	330	53.7	57.5		
Age	619			7.777	0.005*
< 65 years	259	41.8	61.6		
≥ 65 years	360	58.2	53.4		
Dukes’ stage	619			293.83	<0.001*
A	91	14.7	90.3		
B	222	35.9	77.7		
C	161	26.0	51.1		
D	145	23.4	8.7		
Differentiation (WHO grade)	618			18.982	<0.001*
1	19	3.1	83.1		
2	406	65.7	60.9		
3	165	26.7	46.3		
4	28	4.5	41.3		
Histologic type	619			1.311	0.252
adenocarcinoma	539	87.1	58.1		
mucinous carcinoma	80	12.9	48.6		
Tumour location	616			2.126	0.145
Colon	339	55.0	58.3		
Rectum	277	45.0	55.4		
missing	3				

* = p-value significant.

In Cox stepwise multivariate analysis, age (p < 0.001), Dukes’ stage (p < 0.001), location (p = 0.016), and differentiation (p = 0.005) were all independent prognostic factors (Table 3). In Dukes’ B tumours, MMP-9 positivity was an independent prognostic factor (p = 0.034), as was tumour location (p = 0.041).

**Table 3:** Cox stepwise multivariate regression analysis of prognostic factors in 558 colorectal cancer patients (MMP-9)

**Covariate**	**Wald statistic**	**p-value**	**RH**	**95% CI**
Age	31.209	<0.001	1.031	1.020-1.043
Dukes’ stage	299.615	<0.001		
A				
B	2.397		1.664	0.874-3.166
C	27.913		5.274	2.846-9.773
D	117.503		30.446	16.417-56.464
Differentiation (WHO Grade)	12.502	0.005		
1				
2	2.246		2.143	0.791-5.806
3	4.577		3.031	1.097-8.373
4	6.353		4.293	1.383-13.330
Tumour location in rectum	5.815	0.016	1.373	1.061-1.776
Histologic type		NS		
MMP9		NS		

NS = not significant, RH = relative hazard, CI = confidence interval at 95% level.

In the validation series, 5-year survival was 64.8% in MMP-9 positive patients compared to 63.5% in those MMP-9 negative, (p = 0.418). In subgroup analysis of Dukes’ stages there were no significant differences in survival according to stage.

Neither MMP-2 immunoexpression (p = 0.477) nor MMP-8 immunoexpression (p = 0.719) associated in univariate analysis with prognosis.

## Discussion

It is clinically relevant to identify a prognostic marker in Dukes’ B (stage II) disease. This stage is classified as local disease, but 20% will still die from this, with surgery curing 80%. We urgently need biomarkers to identify those 20% at risk in order to offer them adjuvant treatment. Here we show that MMP-9 tumour expression is an independent prognostic factor in Dukes’ B colorectal cancer. In the other stage groups, no significant difference in survival emerged. In Dukes’ B, the difference was so great, that it affected analysis of the whole cohort. In Dukes’ B and C of the validation series, we noticed poorer prognosis among patients with MMP-9 negative tumours, but the differences were not significant.

The overall survival was better in the validation series. The difference in survival may have several reasons. First, pathologic routines have changed and more lymph nodes are nowadays evaluated, which may lead to stage migration. Second, the surgical technique has changed, especially in rectal cancer treatment. Third, the more frequent use of adjuvant treatment might have improved survival.

Our results differ from those in a recent study of stage II CRC in which high MMP-9 expression associated with higher recurrence rate, shorter disease-free survival, and also shorter disease-specific survival, but association with disease-specific survival was not significant in multivariate analysis [21]. In CRC patients, high MMP-9 expression has been associated with liver metastasis [22]. We also analyzed high versus low immunoexpression, but found no association with survival (data not shown). Elevated MMP-9 mRNA levels have been associated with poor disease-free and overall survival [14]. On the other hand, some studies using immunohistochemistry have failed to show any correlation between MMP-9 and survival or clinicopathological parameters [16,23]. In other cancer forms, such as lung cancer, head and neck squamous cell carcinoma, and gastric cancer, MMP-9 associates with poor prognosis [24-26]. Interestingly, in early breast cancer, elevated MMP-9 associates with better prognosis [27].

In cancer, the host response, like the intra- or peritumoral inflammation reaction or desmoplasia, can predict better prognosis [6-8]. Theoretically, some matrix-degrading proteinases may play a defensive role by supporting the immune/inflammatory response. Stromal expression of MMP-9 inversely associates with liver metastasis and tumour infiltration in CRC [28]. Stromal MMP-9 positivity also inhibits metachronous haematogenic metastasis in Dukes’ B and C colorectal cancer patients [29]. Here, we did not evaluate stromal MMP-9 expression, because we used tissue arrays with punches taken from cancerous regions, and our samples were unsuitable for reliable stroma evaluation. The TMA is not as reliable as analysis of whole tissue sections because of intra-tumoural heterogenity of immunohistochemical expression. However, the results of evaluation of TMAs and those of whole tissue sections have been shown to be in concordance [30].

Tumour expression of MMP-2 has been associated with poor prognosis in colorectal [11-13,31], gastric [25], ovarian [32], and breast cancer [33]. Here, we found no correlation between MMP-2 immunoexpression and clinicopathological variables or prognosis of CRC. Results similar to ours have emerged in CRC [34]. Thereby the role of MMP-2 in CRC is still controversial.

In breast cancer, MMP-8 changes metastatic potential *in vitro*[15], and in melanoma and lung cancer it inhibits metastasis formation by modulating cancer cell invasion and adhesion [14]. It was expected that MMP-8 might be a marker for improved prognosis in colorectal cancer, but we noticed no association between MMP-8 immunoexpression and survival.

## Conclusion

The role of MMP-9 immunoexpression in colorectal cancer is dual; it plays a role in matrix degradation enabling tumour invasion, but it also seems to act as a supportive factor for hosts’ defensive mechanisms against cancer spread. Here we show that immunoexpression of MMP-9 is a promising prognostic marker in Dukes’ B (Stage II) CRC, the group of patients for whom we need new markers to identify those at risk who need adjuvant treatment.

## Ethics approval

The study has been approved by the Surgical Ethics Committee of Helsinki University Central Hospital (Dnro HUS 226/E6/06) and National Supervisory Authority for Welfare and Health.

## Abbreviations

BM: Basement membrane; CRC: Colorectal cancer; EMC: Extracellular matrix; H&E: Haematoxylin-eosin; MMP: Matrix metalloproteinase; mRNA: Messenger ribonuclein acid; TMA: Tissue microarray.

## Competing interests

The authors declare that they have no competing interests.

## Authors’ contribution

SK participated in patient data collection, scored samples and, wrote the manuscript. JH scored samples with SK and participated in drafting the manuscript. NL participated in sample analysis. ML participated in statistical analysis. TS provided the MMP-8 antibody and participated in planning and evaluating MMP-8 stainings. JL participated in patient data collection and drafting the manuscript, and performed the statistical analysis. CH conceived the study, participated in its design and co-ordination, was responsible for immunohistochemical stainings and participated in drafting the manuscript. All authors read and approved the final manuscript.

## Pre-publication history

The pre-publication history for this paper can be accessed here:

http://www.biomedcentral.com/1472-6890/12/24/prepub

## References

[B1] Jemal A Bray F Center MM Ferlay J Ward E Forman D Global cancer statistics CA Cancer J Clin 2011 61 2 69 90 10.3322/caac.20107 21296855

[B2] Compton CC Pathology report in colon cancer: what is prognostically important? Dig Dis 1999 17 2 67 79 10.1159/000016908 10545712

[B3] Compton CC Fielding LP Burgart LJ Conley B Cooper HS Hamilton SR Hammond ME Henson DE Hutter RV Nagle RB Nielsen ML Sargent DJ Taylor CR Welton M Willett C Prognostic factors in colorectal cancer. College of American Pathologists Consensus Statement 1999 Arch Pathol Lab Med 2000 124 7 979 994 10888773 10.5858/2000-124-0979-PFICC

[B4] Yang Z Wang L Kang L Xiang J Peng J Cui J Huang Y Wang J Clinicopathologic characteristics and outcomes of patients with obstructive colorectal cancer J Gastrointest Surg 2011 15 7 1213 1222 10.1007/s11605-011-1563-1 21584821

[B5] Anwar MA D’Souza F Coulter R Memon B Khan IM Memon MA Outcome of acutely perforated colorectal cancers: experience of a single district general hospital Surg Oncol 2006 15 2 91 96 10.1016/j.suronc.2006.09.001 17049848

[B6] Crispino P De Toma G Ciardi A Bella A Rivera M Cavallaro G Polistena A Fornari F Unim H Pica R Cassieri C Mingazzini PL Paoluzi P Role of desmoplasia in recurrence of stage II colorectal cancer within five years after surgery and therapeutic implication Cancer Invest 2008 26 4 419 425 10.1080/07357900701788155 18443963

[B7] Galon J Costes A Sanchez-Cabo F Kirilovsky A Mlecnik B Lagorce-Pages C Tosolini M Camus M Berger A Wind P Zinzindohoue F Bruneval P Cugnenc PH Trajanoski Z Fridman WH Pages F Type, density, and location of immune cells within human colorectal tumors predict clinical outcome Science 2006 313 5795 1960 1964 10.1126/science.1129139 17008531

[B8] Ropponen KM Eskelinen MJ Lipponen PK Alhava E Kosma VM Prognostic value of tumour-infiltrating lymphocytes (TILs) in colorectal cancer J Pathol 1997 182 3 318 324 10.1002/(SICI)1096-9896(199707)182:3<318::AID-PATH862>3.0.CO;2-6 9349235

[B9] Coussens LM Werb Z Matrix metalloproteinases and the development of cancer Chem Biol 1996 3 11 895 904 10.1016/S1074-5521(96)90178-7 8939708

[B10] Hasty KA Jeffrey JJ Hibbs MS Welgus HG The collagen substrate specificity of human neutrophil collagenase J Biol Chem 1987 262 21 10048 10052 3038863

[B11] Matsuyama Y Takao S Aikou T Comparison of matrix metalloproteinase expression between primary tumors with or without liver metastasis in pancreatic and colorectal carcinomas J Surg Oncol 2002 80 2 105 110 10.1002/jso.10106 12173379

[B12] Papadopoulou S Scorilas A Arnogianaki N Papapanayiotou B Tzimogiani A Agnantis N Talieri M Expression of gelatinase-A (MMP-2) in human colon cancer and normal colon mucosa Tumour Biol 2001 22 6 383 389 10.1159/000050641 11786732

[B13] Hilska M Roberts PJ Collan YU Laine VJ Kossi J Hirsimaki P Rahkonen O Laato M Prognostic significance of matrix metalloproteinases-1, -2, -7 and −13 and tissue inhibitors of metalloproteinases-1, -2, -3 and −4 in colorectal cancer Int J Cancer 2007 121 4 714 723 10.1002/ijc.22747 17455256

[B14] Zeng ZS Huang Y Cohen AM Guillem JG Prediction of colorectal cancer relapse and survival via tissue RNA levels of matrix metalloproteinase-9 J Clin Oncol 1996 14 12 3133 3140 8955659 10.1200/JCO.1996.14.12.3133

[B15] Karakiulakis G Papanikolaou C Jankovic SM Aletras A Papakonstantinou E Vretou E Mirtsou-Fidani V Increased type IV collagen-degrading activity in metastases originating from primary tumors of the human colon Invasion Metastasis 1997 17 3 158 168 9702942

[B16] Roca F Mauro LV Morandi A Bonadeo F Vaccaro C Quintana GO Specterman S de Kier Joffe EB Pallotta MG Puricelli LI Lastiri J Prognostic value of E-cadherin, beta-catenin, MMPs (7 and 9), and TIMPs (1 and 2) in patients with colorectal carcinoma J Surg Oncol 2006 93 2 151 160 10.1002/jso.20413 16425303

[B17] Gutierrez-Fernandez A Fueyo A Folgueras AR Garabaya C Pennington CJ Pilgrim S Edwards DR Holliday DL Jones JL Span PN Sweep FC Puente XS Lopez-Otin C Matrix metalloproteinase-8 functions as a metastasis suppressor through modulation of tumor cell adhesion and invasion Cancer Res 2008 68 8 2755 2763 10.1158/0008-5472.CAN-07-5154 18413742

[B18] Montel V Kleeman J Agarwal D Spinella D Kawai K Tarin D Altered metastatic behavior of human breast cancer cells after experimental manipulation of matrix metalloproteinase 8 gene expression Cancer Res 2004 64 5 1687 1694 10.1158/0008-5472.CAN-03-2047 14996728

[B19] Davis NC Evans EB Cohen JR Theile DE Staging of colorectal cancer. The Australian clinico-pathological staging (ACPS) system compared with Dukes’ system Dis Colon Rectum 1984 27 11 707 713 10.1007/BF02554593 6499604

[B20] Sorsa T Ding Y Salo T Lauhio A Teronen O Ingman T Ohtani H Andoh N Takeha S Konttinen YT Effects of tetracyclines on neutrophil, gingival and salivary collagenase. A functional and western-blot assessment with special reference to their cellular sources in periodontal disease Ann N Y Acad Sci 1994 732 112 131 10.1111/j.1749-6632.1994.tb24729.x 7978785

[B21] Buhmeida A Bendardaf R Hilska M Collan Y Laato M Syrjanen S Syrjanen K Pyrhonen S Prognostic significance of matrix metalloproteinase-9 (MMP-9) in stage II colorectal carcinoma J Gastrointest Cancer 2009 40 3–4 91 97 19921474 10.1007/s12029-009-9091-x

[B22] Koumura H Sugiyama Y Kunieda K Saji S Significance in gene expression of matrix metalloproteinase-9, urokinase-type plasminogen activator and tissue inhibitor of metalloproteinase for metastases of gastric and/or colo-rectal cancer Gan To Kagaku Ryoho 1997 24 Suppl 2 324 331 9263524

[B23] Jensen SA Vainer B Bartels A Brunner N Sorensen JB Expression of matrix metalloproteinase 9 (MMP-9) and tissue inhibitor of metalloproteinases 1 (TIMP-1) by colorectal cancer cells and adjacent stroma cells–associations with histopathology and patients outcome Eur J Cancer 2010 46 18 3233 3242 10.1016/j.ejca.2010.07.046 20801641

[B24] Brown PD Bloxidge RE Stuart NS Gatter KC Carmichael J Association between expression of activated 72-kilodalton gelatinase and tumor spread in non-small-cell lung carcinoma J Natl Cancer Inst 1993 85 7 574 578 10.1093/jnci/85.7.574 8384265

[B25] Ruokolainen H Paakko P Turpeenniemi-Hujanen T Expression of matrix metalloproteinase-9 in head and neck squamous cell carcinoma: a potential marker for prognosis Clin Cancer Res 2004 10 9 3110 3116 10.1158/1078-0432.CCR-03-0530 15131051

[B26] Sier CF Kubben FJ Ganesh S Heerding MM Griffioen G Hanemaaijer R van Krieken JH Lamers CB Verspaget HW Tissue levels of matrix metalloproteinases MMP-2 and MMP-9 are related to the overall survival of patients with gastric carcinoma Br J Cancer 1996 74 3 413 417 10.1038/bjc.1996.374 8695357 PMC2074643

[B27] Scorilas A Karameris A Arnogiannaki N Ardavanis A Bassilopoulos P Trangas T Talieri M Overexpression of matrix-metalloproteinase-9 in human breast cancer: a potential favourable indicator in node-negative patients Br J Cancer 2001 84 11 1488 1496 10.1054/bjoc.2001.1810 11384099 PMC2363667

[B28] Takeha S Fujiyama Y Bamba T Sorsa T Nagura H Ohtani H Stromal expression of MMP-9 and urokinase receptor is inversely associated with liver metastasis and with infiltrating growth in human colorectal cancer: a novel approach from immune/inflammatory aspect Jpn J Cancer Res 1997 88 1 72 81 10.1111/j.1349-7006.1997.tb00304.x 9045899 PMC5921245

[B29] Saito K Takeha S Shiba K Matsuno S Sorsa T Nagura H Ohtani H Clinicopathologic significance of urokinase receptor- and MMP-9-positive stromal cells in human colorectal cancer: functional multiplicity of matrix degradation on hematogenous metastasis Int J Cancer 2000 86 1 24 29 10.1002/(SICI)1097-0215(20000401)86:1<24::AID-IJC4>3.0.CO;2-A 10728590

[B30] Kallioniemi OP Wagner U Kononen J Sauter G Tissue microarray technology for high-throughput molecular profiling of cancer Hum Mol Genet 2001 10 657 662 10.1093/hmg/10.7.657 11257096

[B31] Masuda H Aoki H Host expression of matrix metalloproteinase-2 and tissue inhibitor of metalloproteinase-2 in normal colon tissue affects metastatic potential of colorectal cancer Dis Colon Rectum 1999 42 3 393 397 10.1007/BF02236360 10223763

[B32] Westerlund A Apaja-Sarkkinen M Hoyhtya M Puistola U Turpeenniemi-Hujanen T Gelatinase A-immunoreactive protein in ovarian lesions- prognostic value in epithelial ovarian cancer Gynecol Oncol 1999 75 1 91 98 10.1006/gyno.1999.5533 10502432

[B33] Talvensaari-Mattila A Paakko P Turpeenniemi-Hujanen T Matrix metalloproteinase-2 (MMP-2) is associated with survival in breast carcinoma Br J Cancer 2003 89 7 1270 1275 10.1038/sj.bjc.6601238 14520459 PMC2394290

[B34] Unsal D Akyurek N Uner A Erpolat OP Han U Akmansu M Mentes BB Dursun A Gelatinase B expression as a prognostic factor in patients with stage II/III rectal carcinoma treated by postoperative adjuvant therapy Am J Clin Oncol 2008 31 1 55 63 10.1097/COC.0b013e318068b4e2 18376229

